# Spondylodiscitis and infective endocarditis: A retrospective cohort analysis of clinical outcomes, microbiological profiles, and mortality in 312 patients

**DOI:** 10.1016/j.bas.2025.105890

**Published:** 2025-11-28

**Authors:** Nicolas Heinz von der Höh, Joanna Maria Przybyl, Philipp Pieroh, Daniel Jurisch, Stefan Glasmacher, Sandra Gräber, Christoph-Eckhard Heyde

**Affiliations:** a Department of Orthopaedics, Trauma and Plastic Surgery, University Hospital Leipzig, Leipzig, Germany; b Cardiology Group Practice for the Diagnosis and Treatment of Heart and Circulatory Diseases in Erfurt, Germany; c University Institute for Medical Microbiology and Virology, Oldenburg, Germany

**Keywords:** Spondylodiscitis, Infective endocarditis, Bacteremia, Echocardiography, Mortality, Spine infection

## Abstract

**Background:**

The coexistence of infective endocarditis (IE) and spondylodiscitis (SD) ranges between 7 % and 30 %, increasing particularly in patients older than 75 years. IE can occur without bacteremia, complicating early diagnosis and therapeutic strategies.

**Objectives:**

To determine the incidence and microbiological spectrum of IE among patients diagnosed and treated with SD, examine the prevalence of associated bacteremia, and evaluate associated clinical outcomes and mortality.

**Methods:**

We conducted a retrospective, monocentric study from January 2016 to December 2020 at a level one spinal surgery center. Included were adult patients (>18 years) with confirmed SD (positive MRI combined with histological and/or microbiological evidence) who underwent echocardiography. Variables analyzed were demographics, localization of SD, microbiological findings, comorbidities, therapeutic approaches, and mortality. Patients were divided into isolated SD and SD with concurrent IE (SD + IE).

**Results:**

Of 312 patients, 31 (9.9 %) had concurrent IE. Bacteremia was documented in 58 % overall but was absent in 16 % of IE cases. Patients with IE had a higher prevalence of coronary artery disease (45 % vs. 26 %; p = 0.0237). Enterococci were significantly more frequent in the IE group (35 % vs. 6 %; p = 0.021). Left heart involvement predominated (80 %), notably the aortic (38.7 %) and mitral valves (29 %). Spinal surgical interventions occurred less frequently in the IE group (45.2 % vs. 85.4 %). Mortality was significantly increased in IE patients (48.4 % vs. 11.74 %; p = 0.0157).

**Conclusions:**

Concomitant infective endocarditis (IE) significantly increases mortality in patients with spondylodiscitis (SD), even in the absence of bacteremia. Routine echocardiographic screening (TTE/TEE) must be standard, as its omission risks missed diagnoses. Interdisciplinary collaboration is essential for timely diagnosis, coordinated treatment, and improved outcomes.

## Highlights

• Coexistence of endocarditis in 9.9 % of spondylodiscitis cases.
• Enterococcal infections significantly more frequent in IE group (35 % vs. 6 %, p = 0.021).
• In-hospital mortality was significantly higher in IE patients (48.4 %, 15/31) compared to SD-only patients (11.7 %, 33/281).
• 16.1 % of IE cases occurred without detectable bacteremia (5/31).
• Echocardiographic screening recommended in all SD patients.

## 1 Introduction

Pyogenic spondylodiscitis is a severe infection of the spine with increasing incidence, particularly among older adults. The rising life expectancy and the growing burden of comorbidities such as diabetes mellitus and chronic kidney disease contribute to a higher susceptibility to infections in the elderly, primarily due to immunosenescence. The infection typically involves the intervertebral disc and adjacent vertebral bodies and is mainly caused by hematogenous spread (Kehrer et al., 2014).

A frequent source of hematogenous dissemination is infective endocarditis (IE), a condition associated with significant morbidity and mortality, with a 30-day mortality rate of approximately 30 %. IE predominantly affects elderly individuals and may develop following the implantation of cardiac devices or intravascular catheters. Notably, spondylodiscitis can occur as a secondary manifestation in up to 10 % of patients with IE (Habib et al., 2015; Haider et al., 2013; Kang et al., 2016).

Recent epidemiological data demonstrate a dramatic increase in the age-specific incidence of spondylodiscitis. In individuals aged 80–89 years, the incidence has reached nearly 50 per 100,000, while in patients over 90 years, it exceeds 3000 per 100,000 (Kramer et al., 2023; Lang et al., 2023). These changes cannot be solely attributed to demographic aging but instead suggest a genuine increase in disease occurrence. This highlights the need for targeted diagnostic and therapeutic approaches for this vulnerable population (Spiegl et al., 2020; Thuny et al., 2012).

Given the substantial mortality and complications associated with both conditions, early and accurate diagnosis is crucial. However, current evidence on the optimal sequence of treatment — whether to address the spinal infection or the cardiac pathology first — remains limited.

The 2023 guidelines of the European Society of Cardiology (ESC) emphasize a more differentiated use of echocardiography in suspected IE (Delgado et al., 2023). Transesophageal echocardiography (TEE) is now strongly recommended not only in cases of prosthetic valves and cardiac devices but also in patients with high clinical suspicion, even if transthoracic echocardiography (TTE) is positive or inconclusive.

The aim of this study was to evaluate the prevalence of IE in patients with confirmed spondylodiscitis, stratified by the presence or absence of bacteremia, and to identify associated risk factors. Particular attention was paid to pathogen profiles, cardiac valve involvement, and mortality outcomes.

## 2 Material and methods

**Fig. 1:**
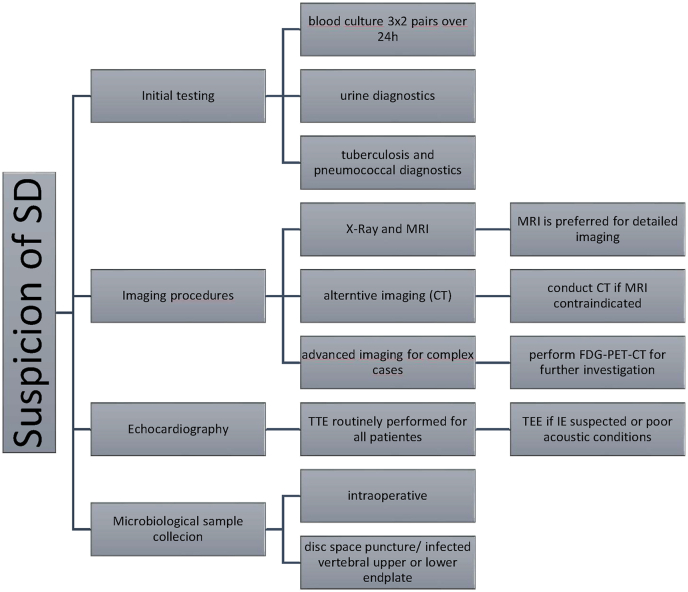
Leipziger diagnostic flowchart if Spondylodiscitis is suspected.

This retrospective observational study included all adult patients (≥18 years) diagnosed and treated for pyogenic spondylodiscitis (SD) at a level one spine center between January 2016 and December 2020. SD was confirmed based on imaging (MRI), microbiological findings (positive culture from intraoperative or CT-guided samples), and/or histopathological evidence. Only patients who underwent transthoracic and/or transesophageal echocardiography (TTE/TEE) as part of routine sepsis and source diagnostics were included (Fig. 1). The diagnosis of concomitant infective endocarditis (IE) was made based on clinical judgment, imaging findings, and interdisciplinary consensus.

Demographic data, comorbidities, spinal infection localization, bacteremia status, microbiological results, cardiac valve involvement, treatment strategies (surgical or conservative), and in-hospital mortality were recorded. Particular attention was given to the timing of cardiac and spinal surgical interventions. Surgical procedures for spondylodiscitis were performed via dorsal, ventral, or combined dorsoventral approaches. The intervention included debridement of the intervertebral disc space, sampling of tissue for microbiological and histological evaluation, extensive irrigation, interbody cage implantation, and supplemental posterior pedicle screw fixation. The interbody cage and disc space was filled with a mixture of allogenic bone, gentamicin, and vancomycin.

All patients received either pathogen-specific antibiotic therapy according to susceptibility testing or empirical therapy based on local hospital guidelines. The minimum duration of antibiotic treatment was six weeks. In cases of infective endocarditis, antibiotic regimens were modified based on recommendations of the institutional cardiology board.

Patients were categorized into two groups: SD without IE and SD with concomitant IE. Comparisons between these groups included microbiological spectrum, distribution of cardiac involvement, and outcome measures.

Statistical analysis was performed using Microsoft Excel and SPSS (version 29). Continuous variables were presented as mean ± standard deviation (SD), and categorical variables as frequencies or percentages. Group comparisons were performed using the chi-square test for categorical variables and Student's t-test or Mann–Whitney *U* test for continuous variables, as appropriate. A p-value of <0.05 was considered statistically significant.

Ethical approval for the study was obtained from the institutional ethics committee (approval no. 566/20-ek).

## 3 Results

A total of 312 patients met the inclusion criteria for the study. Of these, 31 patients (9.9 %) were diagnosed with concomitant infective endocarditis (IE), while 281 patients (90.1 %) had spondylodiscitis (SD) without IE. All 312 patients received transthoracic echocardiography (TTE) as part of the diagnostic protocol (Fig. 1). In all patients with confirmed infective endocarditis (IE; n = 31; 9.9 %), additional transesophageal echocardiography (TEE) was performed following the initial TTE. Consequently, all IE cases were initially suspected based on TTE findings and confirmed by TEE.

Patients in the IE group were older than those in the SD group (mean age 74.4 ± 12.8 years vs. 71.0 ± 12.0 years). Coronary artery disease was documented in n = 14 IE patients (45.2 % of IE) and in n = 75 SD patients (26.7 % of SD) (p = 0.0237). Other comorbidities such as diabetes mellitus (IE: n = 13; 41.9 %; SD: n = 117; 41.6 %), arterial hypertension (IE: n = 27; 87.1 %; SD: n = 224; 79.7 %), chronic kidney disease (IE: n = 15; 48.4 %; SD: n = 121; 43.1 %), and obesity (IE: n = 5; 16.1 %; SD: n = 66; 23.5 %) showed no statistically significant differences. Drug abuse was documented in n = 2 patients in each group (IE: 6.5 %; SD: 0.7 %). All patient characteristics are shown in Table 1.

**Table 1:** Characteristics of patients with and without endocarditis.

Parameter	SD	EC	p- value
No. of patients	281	31	
Mean age, yrs	71+-12	74,4+-12,84	
Females	121	12	
**Spine level**			
*Cervical*	23	2	
*Thoracic*	64	4	
*Thoraco-lumbar*	11	0	
*Lumbar*	168	22	
*Multilevel*	13	3	
**Side Disease**			
***Coronar disease***	**75 (26 %)**	**14 (45 %)**	**0,0237**
*Diabetes mellitus*	117 (41 %)	13 (41 %)	
*Chronic renal failure*	121 (43 %)	15 (48 %)	
*Adipositas*	66 (23 %)	5 (16 %)	
*Arterial hypertension*	224 (79 %)	27 (87 %)	
*Drug abusus*	2 (0,7 %)	2 (6,45 %)	
***Deaths***	**33 (11,74 %)**	**15 (48,4 %)**	**0,0157**

**Fig. 2:**
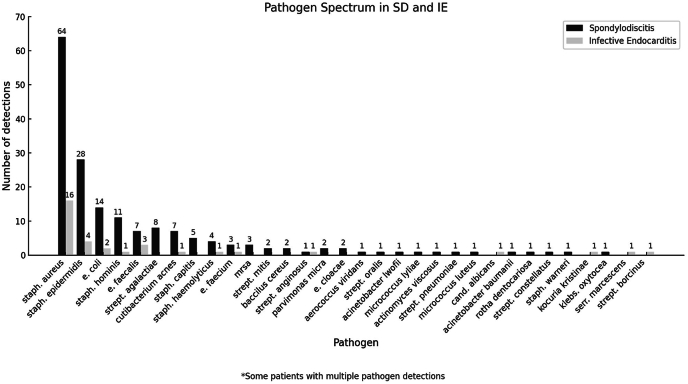
Characteristics of pathogen detections.

Spondylodiscitis was located in the lumbar spine in n = 22 IE patients (71.0 %) and n = 168 SD patients (59.8 %), in the thoracic spine in n = 4 IE (12.9 %) and n = 64 SD patients (22.8 %), in the cervical spine in n = 2 IE (6.5 %) and n = 23 SD patients (8.2 %), and multisegmentally in n = 3 IE (9.7 %) and n = 13 SD patients (4.6 %). Cardiac valve involvement affected the aortic valve in n = 12 patients (38.7 % of IE), the mitral valve in n = 9 patients (29.0 %), and both valves in n = 2 patients (6.4 %). Right heart involvement was observed in n = 6 patients (19.4 %), including n = 3 with pacemaker lead infections (9.7 %) and n = 3 with tricuspid valve infections (9.7 %) (Table 2). Bacteremia was detected in n = 181 patients (58.0 % of total cohort). n = 5 IE patients (16.1 % of IE) had no bacteremia. The majority of bloodstream pathogens were Staphylococci (n = 120; 38.5 %), including n = 104 in the SD group (37.0 %) and n = 16 in the IE group (51.6 %). Streptococci were detected in n = 13 SD patients (4.6 %) and n = 3 IE patients (9.7 %). Enterococci were found in n = 18 SD patients (6.4 %) and n = 11 IE patients (35.5 %) with a statistically significant difference between groups (p = 0.021). Mycobacterial infections were identified in n = 6 patients in the SD group (1.9 %) (Fig. 2). No pathogen with positive histomorphological diagnosed SD was detected in 127 patients (40.7 %). All patients initially received empirical antibiotic therapy, regardless of microbiological confirmation. Once pathogens were identified—either from blood cultures or intraoperative sampling—targeted therapy was initiated based on susceptibility testing. In patients with isolated spondylodiscitis, the average treatment duration was six weeks, with individual adjustments depending on clinical factors. In contrast, patients with concomitant infective endocarditis typically received antibiotic therapy for a median duration of nine weeks, often extended by an additional six weeks in cases with complex cardiac involvement. Spinal surgery was performed in n = 254 patients (81.4 % of total cohort) (IE: n = 14; 45.2 %; SD: n = 240; 85.4 %). Cardiac surgery was performed in n = 5 IE patients (16.1 %). Conservative treatment for IE was pursued in n = 26 patients (83.9 %).

**Table 2:** Characteristics of heart valve or heart device infections.

Parameter	EC
***Left heart***	**25 (80 %)**
*Aortic*	12 (38,7 %)
*Mitral*	9 (29 %)
*Aortic valve replacement*	2 (6,4 %)
*double valve*	2 (6,4 %)
***Right heart***	**6 (20 %)**
*tricuspidal*	3 (9,6 %)
*pulmonary*	0
*Pacemaker electrode*	3 (9,6 %)
*Three valves*	1 (3,22 %)

Overall, n = 48 patients died during hospitalization (15.4 % of total). Of these, n = 15 were in the IE group (48.4 %) and n = 33 in the SD group (11.7 %), resulting in a significantly higher mortality in the IE group (p = 0.0157).

Footnotes for Table 1, Table 2.

– SD: Spondylodiscitis
– EC: Endocarditis
– n: absolute number of patients
– (%): percentage of patients within the respective group
– p < 0.05: statistically significant difference between SD and IE groups

## 4 Discussion

Pyogenic spondylodiscitis is increasingly recognized as a clinically significant spinal infection with rising incidence, particularly in older patients. Despite this trend, the actual prevalence of concomitant infective endocarditis (IE) is likely underestimated due to the absence of standardized cardiac screening protocols. Current guidelines do not mandate routine echocardiographic evaluation in all SD patients, potentially missing clinically silent IE with high associated mortality. Based on our findings, a systematic implementation of echocardiography in SD diagnostics should be considered, and its inclusion in future guideline revisions is strongly recommended. Close interdisciplinary collaboration between spine surgery, cardiology, and infectious disease teams remains essential for improving outcomes in this high-risk patient population.

This study presents data on the coexistence of infective endocarditis (IE) and spondylodiscitis (SD). A total of 312 patients with SD and completed echocardiographic diagnostics were treated over a 5-year period at our institution. To our knowledge, this constitutes one of the largest cohorts analyzing this dual pathology. Co-occurrence of both infections has been previously reported in several cardiology-based investigations (Morelli et al., 2001; Pigrau et al., 2005; Ninet et al., 1984; Weber et al., 2022).

In our study, infective endocarditis (IE) was diagnosed in 31 of 312 patients with spondylodiscitis (SD), corresponding to a prevalence of 9.9 %. This finding is consistent with the data reported by Viezens et al., who identified concomitant IE in 11.0 % of patients with SD in a prospective single-center analysis. In their study, most cases of IE were only detected by transesophageal echocardiography (TEE), while transthoracic echocardiography (TTE) alone was insufficient. This supports the assumption that the true incidence of IE may be underestimated when TEE is not routinely performed (Behmanesh et al., 2019). The lower detection rate compared to the study by Pigrau et al. (2005) may be due to differences in diagnostic strategy. In our cohort, transthoracic echocardiography (TTE) was the standard modality, and transesophageal echocardiography (TEE) was reserved for selected indications. As shown in prior studies (Bai et al., 2017; Bonzi et al., 2018; De Castro et al., 2000), the sensitivity of TTE is limited, and diagnostic yield increases significantly with TEE. Behmanesh et al. (2019) reported an increase in IE detection from 3 % to 30 % after switching to routine TEE.

Compared to Weber et al. (2024), the prevalence of co-infection appears higher in our cohort. This may reflect the clinical setting: as a spine center, we routinely evaluate patients with back pain, while cardiology units may not systematically screen for SD, leading to underestimation of co-occurrence.

Blood cultures were negative in 5 of 31 IE cases (17 %). This corresponds with previous reports indicating that up to 27 % of IE cases may present without bacteremia (Kini et al., 2010). Notably, spondylodiscitis has been newly included as a minor criterion in the revised 2023 Duke criteria for IE diagnosis. Based on our data and supported by current literature, we recommend echocardiographic screening in all patients with SD, regardless of bacteremia status. The absence of bacteremia in several cases could not be attributed to prior antibiotic exposure.

Regarding risk factors, patients with IE more frequently exhibited coronary artery disease, which aligns with prior studies (Behmanesh et al., 2019; Carbone et al., 2020). Other comorbidities, including diabetes mellitus, renal insufficiency, hypertension, and obesity, did not differ significantly.

The microbiological analysis revealed a significant association between enterococcal infections and the presence of IE. Enterococci were more frequently isolated in the IE group (n = 11) than in the SD group (n = 18; p = 0.021). This observation corresponds with results from Yagdiran et al. (2022) and is consistent with other IE-focused studies (Carbone et al., 2020; Del Pace et al., 2021; Weber et al., 2024). Staphylococci remained the most frequently isolated pathogens overall (Yagdiran et al., 2022).

Valve involvement predominantly affected the aortic valve (n = 12; 38.7 %), which is consistent with current literature (Saha et al., 2023; Cahill et al., 2016).

Surgical decision-making in IE patients was guided by interdisciplinary cardiology board recommendations. In our cohort, SD was surgically addressed first. In our cohort, the observed in-hospital mortality among patients undergoing cardiac surgery was 20 %, which is considerably higher than the 8 % typically reported in isolated IE cases. This discrepancy may be explained by the complexity of dual pathology, as patients with both spondylodiscitis and endocarditis often present with multiple comorbidities and advanced infection stages. Kang et al. demonstrated in the EASE trial that even in selected IE patients undergoing early surgery, long-term mortality rates remained around 8 % at 4 years, but outcomes were dependent on strict surgical indications and patient selection [KCJ 2016]. In contrast, our cohort likely included higher-risk patients, such as those with delayed diagnosis or advanced structural involvement, which may explain the elevated surgical mortality. In contrast, Del Pace et al. [BMC Cardiovasc Disord 2021] observed no increased mortality in patients with coexisting SD and IE, suggesting that the presence of SD alone may not worsen prognosis if timely diagnosed and appropriately managed. However, our cohort showed a significant mortality difference, emphasizing the impact of initial clinical presentation, surgical sequence, and institutional treatment standards.

Overall, the presence of IE increased in-hospital mortality among SD patients from 11.7 % to 48.4 % (p = 0.0157). In contrast, Viezens et al. (2022) reported lower mortality in the IE group, potentially due to the exclusive use of conservative management in that cohort.

Moreover, Viezens et al. demonstrated that the presence of IE significantly delayed surgical treatment and prolonged hospital stay, despite similar mortality rates. Our findings corroborate these results and emphasize the importance of early, standardized interdisciplinary diagnostics in patients with SD.

Based on our findings, echocardiographic screening—initially via transthoracic echocardiography (TTE) and followed by transesophageal echocardiography (TEE) when indicated—should be mandatory in all patients with confirmed spondylodiscitis. Omitting cardiac imaging risks missing clinically silent IE, which may result in fatal outcomes. Given the complexity and overlap of spinal and cardiac infections, timely and structured collaboration between spine surgeons, cardiologists, and infectious disease specialists is not optional but essential for diagnostic accuracy and optimal patient outcomes.

### 4.1 Limitations

First, it was conducted retrospectively and in a single tertiary care center, which may introduce referral and selection bias and limit the generalizability of the findings. Second, the use of transthoracic echocardiography (TTE) as the initial diagnostic modality in all patients may have led to underdiagnosis of clinically silent or early-stage endocarditis, especially in the absence of bacteremia. The decision to perform transesophageal echocardiography (TEE) was made selectively and not standardized, which likely introduced heterogeneity in diagnostic sensitivity. Third, the observational nature of the analysis precludes causal inferences between treatment strategy and outcome, and confounding variables could not be fully controlled. Fourth, data on timing, sequence, and rationale for operative interventions were extracted from chart review and may be subject to documentation bias. Lastly, the relatively small size of the infective endocarditis subgroup limited the statistical power of comparisons and precluded multivariable risk adjustment for mortality. Furthermore, data on systemic inflammatory response, sepsis status, early in-hospital mortality (e.g., within 14 days), and length of hospital stay (LOS) were not uniformly available due to limitations in the retrospective documentation. As a result, subgroup analyses regarding septic burden and healthcare resource utilization could not be performed and remain a relevant focus for future prospective studies.

## 5 Conclusions

Our data demonstrate a markedly increased in-hospital mortality in patients with concomitant infective endocarditis (IE) compared to those with isolated spondylodiscitis (48.4 % vs. 11.7 %). While we were unable to systematically assess delays in cardiac surgical intervention, the high mortality rate in the IE group likely reflects the cumulative burden of comorbidities, advanced systemic infection, and potential limitations in therapeutic response. This aligns with findings from Viezens et al., who also reported elevated mortality in co-infected patients, particularly when surgical intervention was deferred or not performed (15). In contrast, Del Pace et al. observed no significant mortality increase in patients with SD + IE, potentially due to earlier diagnosis and standardized management pathways (9). Kang et al. further highlighted the prognostic relevance of early surgical intervention in IE. Taken together, our data underscore the prognostic impact of timely and coordinated interdisciplinary care in these complex patients (23).

Concomitant infective endocarditis in patients with spondylodiscitis represents a clinically relevant condition associated with increased mortality, especially in cases without bacteremia. Routine echocardiographic screening should be considered in all patients with confirmed spondylodiscitis to enable early diagnosis of cardiac involvement. Early interdisciplinary coordination between spine surgeons, cardiologists, and infectious disease specialists is essential to optimize outcomes in this vulnerable patient population.

## Declaration of competing interest

All other authors declare no conflicts of interest.
